# Nomograms for metastasis of non-sentinel lymph nodes or more than three lymph nodes in patients with one or two positive sentinel lymph nodes

**DOI:** 10.3389/fonc.2024.1413936

**Published:** 2024-05-21

**Authors:** Xue-Er Wang, Zhao Bi, Jin Zhang, Yong-Sheng Wang

**Affiliations:** ^1^ The Third Department of Breast Cancer, Tianjin Medical University Cancer Institute and Hospital, National Clinical Research Center for Cancer, Tianjin, China; ^2^ Key Laboratory of Breast Cancer Prevention and Therapy, Tianjin Medical University Cancer Institute and Hospital, Tianjin, China; ^3^ Tianjin's Clinical Research Center for Cancer, Tianjin Medical University Cancer Institute and Hospital, Tianjin, China; ^4^ Shandong Cancer Hospital and Institute, Shandong First Medical University and Shandong Academy of Medical Sciences, Jinan, China

**Keywords:** breast cancer, sentinel lymph node biopsy, nomogram, internal mammary lymph node, regional nodal irradiation

## Abstract

**Purpose:**

The purpose of this study was to provide advice for the indication of regional nodal irradiation (RNI) in patients with one to two positive sentinel lymph nodes (SLNs) without axillary lymph node dissection (ALND).

**Methods:**

We conducted a retrospective study in Shandong Cancer Hospital, Fudan University Shanghai Cancer Center, and West China Hospital. Logistic analysis was performed in order to explore the influencing factors of positive non-SLNs (NSLNs) and >3 positive nodes among patients with one to two SLNs+. Then, nomograms were constructed.

**Results:**

Between May 2010 and 2020, among the 2,845 patients with one to two SLNs+ undergoing ALND (1,992 patients in the training set and 853 patients in the validation set), there were 34.3% harbored NSLNs+ and 15.6% harbored >3 positive nodes. Multivariate analysis showed that cN stage, the number of positive/negative SLN, pathological tumor stage, lympho-vascular invasion (LVI), multicenter, and molecular subtypes were significantly associated with NSLN metastasis. Similarly, multivariate analysis also showed that cN stage, the number of positive/negative SLNs, pathological tumor stage, and LVI could be independent predictors of >3 positive nodes. Then, nomograms for NSLN metastasis and >3 positive nodes were constructed using these parameters, respectively.

**Conclusions:**

The nomograms will be useful in estimating positive NSLNs and >3 positive nodes, and they might provide advice for the optimization of RNI.

## 1 Introduction

The health burden of cancer is increasing in China, with more than 1.6 million people being diagnosed and 1.2 million people dying of the disease each year. As in most other countries, breast cancer is now the most common cancer in Chinese women (1). In the past, the regional nodal irradiation (RNI) was based on axillary tumor burden information after axillary lymph node dissection (ALND) in breast cancer. The evidence-based medical evidence has supported that sentinel lymph node biopsy (SLNB) followed by RNI could replace safely ALND in patients with limited sentinel lymph node (SLN) involvement. The optimization of RNI fields should also take into account this newer approach in clinical practice (2). The Early Breast Cancer Trialists' Collaborative Group (EBCTCG) meta-analysis showed that RNI in patients with positive axillary lymph nodes (ALNs) could improve survival, even after ALND (3). However, there was still no related evidence to design the optimal RNI fields in patients with one to two SLNs+ without ALND.

There were 15.9%–38.6% of patients with positive non-SLNs (NSLNs) when detected one to two SLNs+ (4–7); in other words, there might be one-third of patients with one to two SLNs+ without ALND that have additional axilla up-stage (8). So, the RNI fields of these patients should not be smaller than patients with pN1 after ALND (8).

The purpose of this study was to identify the predict factors of axilla residual tumor burden in patients with one to two SLNs+ based on multicenter population data. Then, we created nomograms that could predict axilla residual tumor burden in patients with one to two SLNs+ without ALND, in order to provide advice for the optimization of RNI, including internal mammary nodal irradiation (IMNI).

## 2 Patients and methods

### 2.1 Patients’ characteristics

Between May 2010 and 2020, we enrolled patients with breast cancer in Shandong Cancer Hospital, Fudan University Shanghai Cancer Center, and West China Hospital of Sichuan University. The inclusion criteria include the following: 1) histologically confirmed invasive breast cancer; 2) cN0 on physical examination or imaging abnormal with/without confirmatory biopsy. According to the National Comprehensive Cancer Network (NCCN) and American Society of Clinical Oncology (ASCO) guidelines, patients with imaging abnormal disease can be offered SLNB as first-line axillary staging (1, 9); 3) undergone SLNB followed by ALND; and 4) had one to two SLNs+. The exclusion criteria include the following: 1) T3–4 primary tumor; 2) bilateral breast cancer; 3) undergone neoadjuvant therapy; and 4) received axillary surgery or radiotherapy. We collected the clinico-pathological data of enrolled patients.

The informed consent had been agreed by the ethical committee (No. SDTHEC20220324) of Shandong Cancer Hospital. The study protocol was approved by the Institutional Review Board of the Shandong Cancer Hospital, and the study was performed in accordance with the principles of the Declaration of Helsinki.

### 2.2 Surgery

Each center detected SLNs according to the same method. The SLNB was done with technetium-99m colloid and blue dye. All radioactive or blue-stained ALNs were detected as SLNs. The ALND was defined as a dissection of at least ten nodes from anatomical levels I and II (10). Each SLN was examined at multiple histologic levels (11).

### 2.3 Pathological evaluation

Before treatment, all patients were biopsied by ultrasound. The pathological evaluation including Hematoxylin-Eosin (HE) and Immunohistochemical (IHC) staining. Positive hormone receptor (HR) status was defined as at least 1% of tumor cells expressing the receptor. HER-2 status was determined on the basis of the ASCO/College of American Pathologists guidelines. To accurately evaluate the effect of molecular subtypes, we classified patients into three subtypes: HR-positive/HER-2–negative (HR+/HER2−), triple-negative (TNBC), and HER-2–positive (HER2+) subtype.

### 2.4 Statistical analysis

We analyzed the correlation between clinic-pathological factors and ALN status. Univariate analysis was performed using the Pearson chi-square or Fisher exact test. Multivariable logistic regression analysis was performed using backward stepwise analysis. Then, nomogram was constructed by “rms” package for R. We used SPSS software (statistics 22.0) and R software (version 3.3.3) to perform statistical analysis. A *p* < 0.05 was considered statistically significant.

## 3 Results

**Figure 1:**
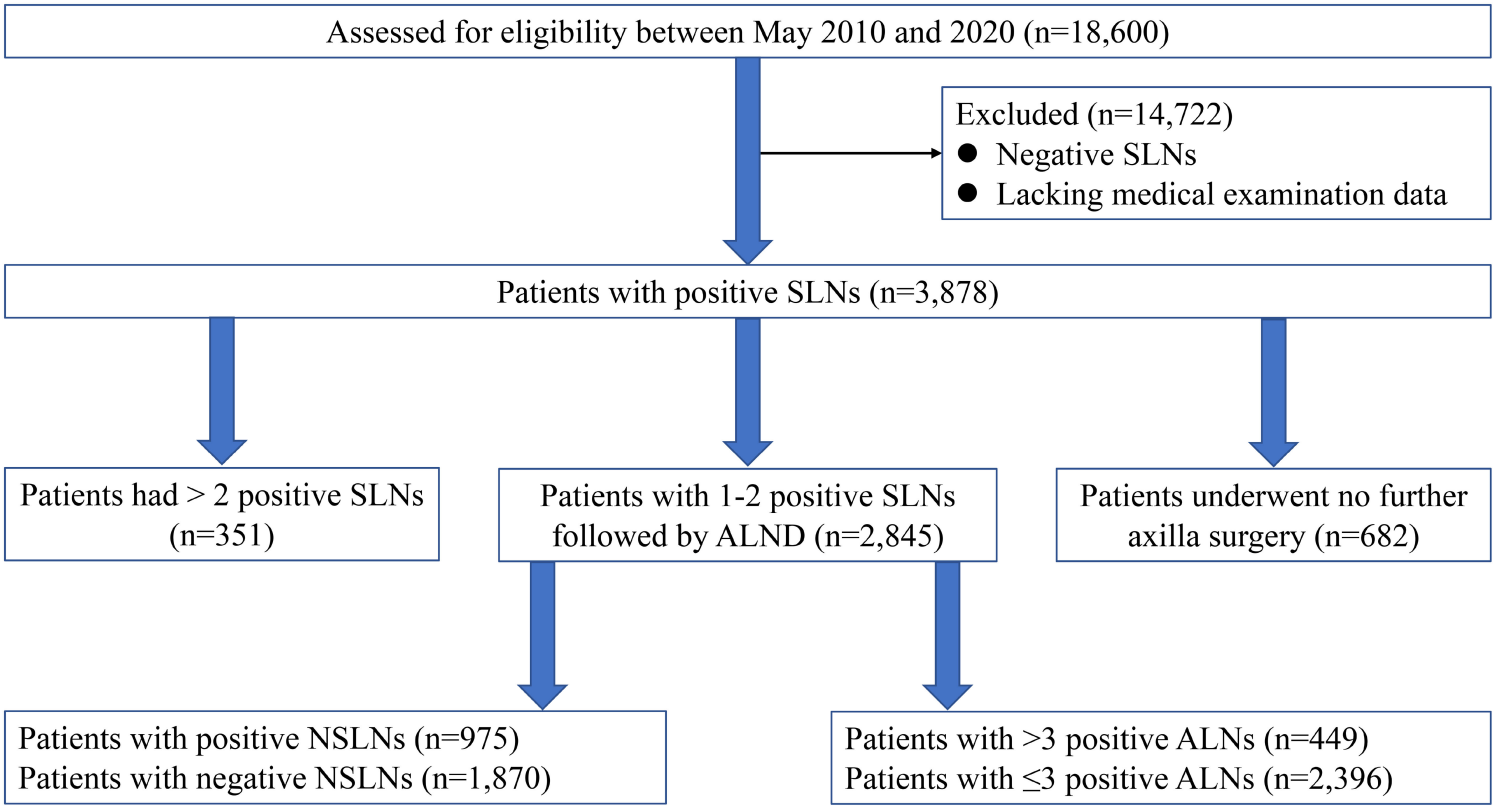
The consort diagram of the trial.

**Figure 1** shows the consort diagram of this study. Between May 2010 and 2020, a total of 18,600 patients with breast cancer who underwent SLNB were identified on the basis of the database of the three institutions. After excluding cases having negative SLNs or lacking medical examination data, 2,845 patients with one to two SLNs+ followed by ALND were finally enrolled in Shandong Cancer Hospital (n = 556), Fudan University Shanghai Cancer Center (n = 1,800), and West China Hospital (n = 489).

We used computer to create a unique, random number for each enrolled patient. Patients were classified in line with the random numbers. Finally, 1,992 patients were designated as the training set, and the other 853 patients were designated as the validation set. **Table 1** shows the clinical-pathologic characteristics of enrolled patients. The median age was 48 years (range of 21–80 years). Notably, there were 84.2%, 11.8%, and 4.0% of patients had one to three positive nodes (pN1), four to nine positive nodes (pN2), and more than nine positive nodes (pN3), respectively.

**Table 1:** The clinical-pathologic characteristics of 2,845 patients.

Characteristics	No. of patients (%)		
	Training set, n = 1,992	Validation set, n = 853	Total, n = 2,845
Age, median (range), years	48 (21–82)	47 (21–82)	48 (21–82)
Tumor size, median (range), cm	2.1 (0.1–5.0)	2.0 (0.1–4.8)	2.1 (0.1–8.0)
Pathological tumor stage			
pT1	980 (49.2%)	426 (49.9%)	1,406 (49.4%)
pT2	1,012 (50.8%)	427 (50.1%)	1,439 (50.6%)
Axillary lymph node metastasis			
1–3	1,672 (83.9%)	724 (84.9%)	2,396 (84.2%)
4–9	253 (12.7%)	96 (11.2%)	349 (12.3%)
>9	67 (3.4%)	33 (3.9%)	100 (3.5%)
Positive SLN+			
1	1,397 (70.1%)	607 (71.2%)	2,004 (70.4%)
2	595 (29.9%)	246 (28.8%)	841 (29.6%)
Negative SLN+			
0	257 (12.9%)	113 (13.2%)	371 (13.0%)
1	418 (21.0%)	168 (19.7%)	585 (20.6%)
2	534 (26.8%)	227 (26.6%)	761 (26.7%)
3	421 (21.1%)	177 (20.8%)	598 (21.1%)
4	222 (11.1%)	100 (11.7%)	322 (11.3%)
>4	140 (7.0%)	68 (8.0%)	208 (7.3%)
Imaging abnormal nodes			
cN0	1,958 (98.3%)	844 (98.9%)	2,802 (98.5%)
iN+	34 (1.7%)	9 (1.1%)	43 (1.5%)
Tumor type			
Ductal, I	40 (2.0%)	16 (1.9%)	52 (1.9%)
Ductal, II	1,151 (57.8%)	484 (56.7%)	1,635 (57.5%)
Ductal, III	662 (33.2%)	298 (34.9%)	960 (33.7%)
Lobular	73 (3.7%)	31 (3.6%)	104 (3.7%)
Special	66 (3.3%)	24 (2.8%)	90 (3.2%)
Lymph-vascular invasion			
Yes	910 (45.7%)	382 (44.8%)	1,292 (45.4%)
No	1,082 (54.3%)	471 (55.2%)	1,553 (54.6%)
Molecular subtypes			
HR+/HER2−	1,273 (63.9%)	544 (63.8%)	1,817 (63.9%)
HER2+	536 (26.9%)	215 (25.2%)	751 (26.4%)
TN	183 (9.2%)	94 (11.0%)	277 (9.7%)
Type of breast surgery			
BCS	422 (21.2%)	199 (23.3%)	621 (21.8%)
Mastectomy	1,570 (78.8%)	654 (76.7%)	2,224 (78.2%)
Multifocal/multicenter			
Yes	147 (7.4%)	65 (7.6%)	212 (7.5%)
No	1,845 (92.6%)	788 (92.4%)	2,633 (92.5%)

SLN, sentinel lymph node; HR+/HER2−, hormone receptor positive/HER-2 negative; HER2+, HER-2 positive; TN, triple negative.

The number of positive SLNs and NSLNs is summarized in **Table 2**. Among the 2,845 patients with one to two SLNs+, 34.3% (975) of them had positive NSLNs, whereas the remaining 65.7% had negative pathological NSLNs. Out of 2,845 patients with one to two SLNs+, 15.8% (449) of them had >3 metastatic ALNs, whereas the remaining 84.2% had ≤3 positive ALNs.

**Table 2:** Association between positive SLNs and NSLNs in the whole population.

Pathological positive SLNs	Pathological positive NSLNs				
	0	1	2	≥ 3	Total
1	1,433 (71.5%)	277 (13.8%)	103 (5.2%)	191 (9.5%)	2,004 (100.0%)
2	437 (51.9%)	146 (17.4%)	80 (9.5%)	178 (21.2%)	841 (100.0%)
Total	1,870 (65.7%)	423 (14.9%)	183 (6.4%)	369 (13.0%)	2,845 (100.0%)

SLN, sentinel lymph node; NSLN, non-sentinel lymph node.

The logistic regression analysis results are shown in **Table 3**. The multivariate analysis indicated that the independent predictors of positive NSLNs including clinical nodal stage (OR = 2.841; 95% CI, 1.487–5.430; *p* = 0.002), the number of positive SLNs (OR = 1.737; 95% CI, 1.451–2.079; *p* < 0.001), the number of negative SLNs (OR = 0.722; 95% CI, 0.678–0.770; *p* < 0.001), pT stage (OR = 1.204; 95% CI, 1.017–1.425, *p* = 0.031), multicenter (OR = 1.636; 95% CI, 1.195–2.239; *p* = 0.002), lympho-vascular invasion (LVI; OR = 3.564; 95% CI, 3.000–4.234; *p* = 0.004), and molecular subtypes (OR = 0.826; 95% CI, 0.726–0.940, *p* = 0.004).

**Table 3:** Clinicopathologic characteristics association with axilla tumor burden.

Characteristic	Negative NSLNs (n = 1,870)	Positive NSLNs (n = 975)	Multivariable analysis	1–3 positive total ALNs (n = 2,396)	>3 positive total ALNs (n = 449)	Multivariable analysis
			*p*-value			*p*-value
Pathological T stage			0.031			0.027
pT1	965 (51.6%)	441 (45.2%)		1,222 (51.0%)	184 (41.0%)	
pT2	905 (48.4%)	534 (54.8%)		1,174 (49.0%)	265 (59.0%)	
Clinical nodal stage			0.002			0.001
cN0	1,851 (98.9%)	951 (97.5%)		2,367 (98.8%)	435 (96.8%)	
iN+	19 (1.1%)	24 (2.5%)		29 (1.2%)	14 (3.2%)	
Positive SLNs			<0.001			<0.001
1	1,433 (76.6%)	571 (58.6%)		1,813 (75.7%)	191 (42.5%)	
2	437 (23.4%)	404 (41.4%)		583 (24.3%)	258 (57.5%)	
Negative SLNs			<0.001			<0.001
0	169 (9.0%)	201 (20.6%)		234 (9.7%)	136 (30.3%)	
1	346 (18.5%)	240 (24.6%)		471 (19.6%)	115 (25.6%)	
2	515 (27.5%)	246 (25.3%)		659 (27.6%)	102 (22.7%)	
3	427 (22.8%)	171 (17.5%)		531 (22.3%)	67 (14.9%)	
4	247 (13.2%)	75 (7.7%)		303 (12.6%)	19 (4.2%)	
>4	166 (9.0%)	42 (4.3%)		198 (8.2%)	10 (2.3%)	
Tumor type						
Ductal, I	45 (2.4%)	11 (1.1%)		52 (2.2%)	4 (0.9%)	
Ductal, II	1,067 (57.1%)	568 (58.3%)		1,394 (58.2%)	241 (53.7%)	
Ductal, III	631 (33.7%)	329 (33.8%)		794 (33.1%)	166 (36.9%)	
Lobular	63 (3.4%)	41 (4.2%)		79 (3.3%)	25 (5.6%)	
Special	64 (3.4%)	26 (2.6%)		77 (3.2%)	13 (2.9%)	
LVI			<0.001			<0.001
No	1,208 (64.6%)	345 (35.4%)		1,427 (59.6%)	126 (28.1%)	
Yes	662 (35.4%)	630 (64.6%)		969 (40.4%)	323 (71.9%)	
Molecular subtypes			0.004			
HR+/HER2−	1,172 (62.7%)	645 (66.2%)		1,530 (63.8%)	287 (63.9%)	
HER-2+	492 (26.3%)	259 (25.6%)		625 (26.1%)	126 (28.1%)	
TN	206 (11.0%)	71 (7.2%)		241 (10.1%)	36 (8.0%)	
Multifocal/multicenter			0.002			
No	1,751 (93.6%)	882 (90.5%)		2,220 (92.6%)	413 (92.0%)	
Yes	119 (6.3%)	93 (9.5%)		176 (7.4%)	36 (8.0%)	

SLN, sentinel lymph node; ALN, axillary lymph node; HR+/HER2−, hormone receptor positive/HER-2 negative; HER2+, HER-2 positive; TN, triple negative; LVI, lymph-vascular invasion.

**Figure 2:**
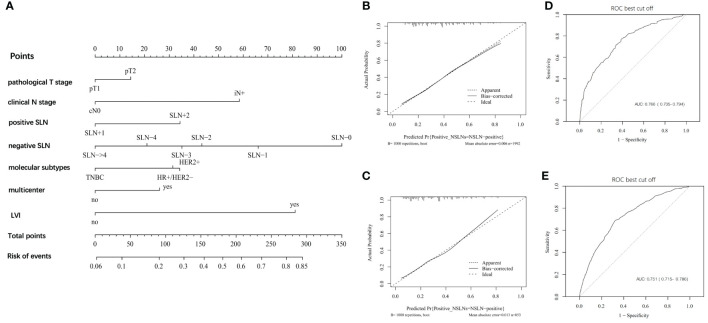
The development and validation of nomogram to predict patients with positive NSLNs. **(A)** The nomogram to predict patients with positive NSLNs in population with one to two positive SLNs. To calculate the probability of positive NSLNs, the scores for each factor were summed up. In addition, the total scores and bottom risk scale were referenced. **(B)** The receiver operating characteristic (ROC) curve in the training cohort indicates an AUC value of 0.766. **(C)** In the validation cohort, the ROC curve indicates an AUC of 0.751. **(D)** The calibration curve showed a satisfactory fit between the observed and predicted outcomes in the training cohorts. **(E)** The calibration curve in the validation cohorts.

According to results of multivariate analysis, a nomogram was constructed to predict positive NSLNs in patients with one to two SLNs+ (**Figure 2A**). The prediction accuracy of different cutoff point is shown in **Supplementary Table 1**. In the training set, the area under the curve (AUC) value was 0.766 (95% CI, 0.735–0.794) (**Figure 2B**). In the external validation set, the AUC value was 0.751 (95% CI, 0.715–0.786), showing a good discriminatory ability (**Figure 2C**). The difference between the two AUCs was not statistically significant (*p* = 0.423). The calibration curve showed a satisfactory fit between the observed and predicted outcomes in the training sets (**Figure 2D**) and validation sets (**Figure 2E**).

**Figure 3:**
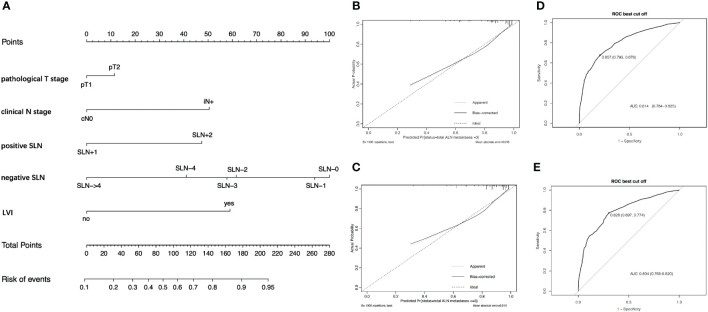
The development and validation of nomogram to predict patients with >3 positive ALNs. **(A)** The nomogram to predict patients with >3 positive ALNs in population with one to two positive SLNs. To calculate the probability of >3 positive ALNs, the scores for each factor were summed up. In addition, the total scores and bottom risk scale were referenced. **(B)** The ROC curve in the training cohort indicates an AUC value of 0.814. **(C)** In the validation cohort, the ROC curve indicates an AUC of 0.804. **(D)** The calibration curve showed a satisfactory fit between the observed and predicted outcomes in the training cohorts. **(E)** The calibration curve in the validation cohorts.

**Table 3** also shows the logistic regression analysis of >3 positive nodes. The multivariate analysis indicated that the independent predictors of >3 positive ALNs including the number of SLNs+ (OR = 3.077; 95% CI, 2.460–3.849; *p* < 0.001), the number of negative SLNs (OR = 0.614; 95% CI, 0.561–0.672; *p* < 0.001), pT stage (OR = 1.317; 95% CI, 1.052–1.648; *p* = 0.016), LVI (OR = 4.078; 95% CI, 3.208–5.184; *p* < 0.001), and cN stage (OR = 3.366; 95% CI, 1.639–6.911; *p* = 0.001). Similarly, a nomogram was also created to predict >3 positive ALNs in patients with one to two SLNs+ (**Figure 3A**). The prediction accuracy of different cutoff point was shown in **Supplementary Table 2**. The nomogram had an AUC value of 0.814 (95% CI, 0.784–0.825) in the training set (**Figure 3B**) and 0.804 (95% CI, 0.768–0.820) in the validation set (**Figure 3C**). The calibration curve showed a satisfactory fit between the observed and predicted outcomes in the training sets (**Figure 3D**) and the validation sets (**Figure 3E**).

## 4 Discussion

In the era of SLNB, SLNB combined with radiotherapy replaced ALND might be the preferred strategy in patients with one to two SLNs+. In our study, we also observed that there were 19.3% of patients with one to two SLNs+ who did not receive ALND, and this trend will be greater. The changing perceptions of axilla treatment make it impossible to fully assess the overall ALNs metastases status (12). Moreover, omitting ALND created a new area of uncertainty for RNI in patients with one to two SLNs+ (8, 12). In the era of SLNB, RNI fields need to be designed in the case of limited nodal tumor information. Therefore, the strength of our study was that the nomograms could help to select precisely populations with one or two positive SLNs that would have positive NSLNs or >3 positive nodes on final pathology. They could provide advice for the optimization of RNI in Chinese patients, and it will be of value to surgeons, medical oncologists, radiation oncologist, and patients in discussing treatment options and their potential outcomes. RNI is strongly recommended to reduce the risk of LRR when the risk of NSLN metastasis >30%. Similarly, IMNI is strongly recommended to be performed when the risk of >3 ALN+ exceeds 20%.

At present, the nomograms have been confirmed to predict axilla metastasis status, combined with actual clinical situations (13–17). Based on the multicenter population data, we developed the nomogram to predict NSLN metastasis in patients with one to two SLNs+ without ALND. In patients with high risk of NSLN metastasis, the risk of recurrence is also high. If no further axilla surgery is performed, then the infra/supraclavicular regions irradiation should be performed to further reduce the risk of regional recurrence. This treatment strategy might the safest approach in the era of limited data concerning outcome after SLNB without ALND (12).

Meanwhile, we found that molecular subtype was also an important factor for predicting NSLN metastasis. Compared with patients with TNBC and HR+/HER2−, patients with HER2+ had a higher probability of NSLN metastasis in patients with one to two SLNs+. This observation is consistent with several published series relating these characteristics to molecular subtype. Crabb et al. (18) demonstrated in a retrospective analysis of 3,441 early-stage breast cancers that subtype as approximated by ER, PR, and HER-2 was predictive of nodal involvement, independent of grade and tumor size. The TNBC subtype had the lowest odds of having ALN involvement, with an OR of 0.53 (95% CI, 0.41–0.6; p < 0.0001) relative to the HR+/HER2− subtype. Ugras et al. (19) also found that HR+/HER2− and TNBC tumors were also less likely to have high-volume lymph node involvement (≥4 nodes involved) than HER2+ tumors. It is suggested that molecular subtype could be used as a predictive factor of lymph node metastasis.

IMNI is one of the main managements of internal mammary region (20–22). However, the benefits of IMNI may also be diluted to some extent by increasing local control when systemic therapy is effective. So, it is very important to grasp indication of IMNI accurately (22). At present, number of positive axillary nodes are still the main indication of IMNI, as it is associated with internal mammary node metastasis. The NCCN guidelines recommend that IMNI should be performed with >3 positive ALNs (category 1) and strongly suggest IMNI to patients with 1 to 3 positive ALNs (category 2A) (9). With more patients received SLNB omitting ALND, IMNI must also be completed without axilla tumor burden.

This study had some limitations. First, this retrospective database–based analysis may increase selection bias in the assignment of treatments. Second, our data are ethnically unique population, and the nomogram was not validated on external population. Third, the IMNI should base on the comprehensive judgement in clinical practice, such as tumor location, biological subtyping, and grade. In addition, we will further explore the indication of IMNI.

In summary, the nomogram will be useful in estimating the likelihood of having positive NSLNs and >3 positive nodes. Moreover, we hope our nomograms can provide advice for the optimization of RNI. In the era of SLNB, the benefits of systemic therapy and radiation therapy can be combined to narrow the scope of surgery and reduce complications, ultimately achieving a “net benefit” of breast cancer treatment.

## Data availability statement

The raw data supporting the conclusions of this article will be made available by the authors, without undue reservation.

## Ethics statement

The informed consent had been agreed by the ethical committee (No. SDTHEC20220324) of Shandong Cancer Hospital. The studies were conducted in accordance with the local legislation and institutional requirements. Written informed consent for participation in this study was provided by the participants’ legal guardians/next of kin.

## Author contributions

XW: Conceptualization, Data curation, Formal analysis, Writing – original draft, Writing – review & editing. ZB: Conceptualization, Data curation, Formal analysis, Writing – original draft, Writing – review & editing. JZ: Conceptualization, Formal analysis, Writing – review & editing. YW: Conceptualization, Formal analysis, Funding acquisition, Writing – original draft, Writing – review & editing.
