# Supplementary material for: Nomograms for metastasis of non-sentinel lymph nodes or more than three lymph nodes in patients with one or two positive sentinel lymph nodes
Source: Front Oncol. 2024 May 21;14:1413936. doi: 10.3389/fonc.2024.1413936 (PMC11148251; doi:10.3389/fonc.2024.1413936)
Supplement: Supplementary file 1 [file Table_1.docx]

Supplementary table 1: Classification accuracy of prediction probability (NSLN+) at different risk cutoff points for the model

| Predicted probability(%） | Sensitivity (%) | Specificity (%) | Accuracy (%) |
| --- | --- | --- | --- |
| ≥30% | 78.6% | 37.1% | 75.3% |
| ≥40% | 68.5% | 67.9% | 74.1% |
| ≥50% | 57.5% | 74.5% | 72.9% |
| ≥60% | 39.3% | 87.8% | 63.6% |
