# Supplementary material for: Nomograms for metastasis of non-sentinel lymph nodes or more than three lymph nodes in patients with one or two positive sentinel lymph nodes
Source: Front Oncol. 2024 May 21;14:1413936. doi: 10.3389/fonc.2024.1413936 (PMC11148251; doi:10.3389/fonc.2024.1413936)
Supplement: Supplementary file 2 [file Table_2.docx]

**Supplementary table 2: Classification accuracy of prediction probability (≥3 ALN+) at different risk cutoff points for the model**

| Predicted probability(%） | Sensitivity (%) | Specificity (%) | Accuracy (%) |
| --- | --- | --- | --- |
| ≥20% | 100% | 0% | 84.2% |
| ≥50% | 96.3% | 16.4% | 83.7% |
| ≥75% | 84.8% | 52.3% | 77.4% |
| ≥80% | 79.9% | 64.1% | 73.1% |
| ≥85% | 69.3% | 74.9% | 70.2% |
